# Inhibition of the MEK/ERK pathway augments nab-paclitaxel-based chemotherapy effects in preclinical models of pancreatic cancer

**DOI:** 10.18632/oncotarget.23684

**Published:** 2017-12-25

**Authors:** Niranjan Awasthi, Sheena Monahan, Alexis Stefaniak, Margaret A. Schwarz, Roderich E. Schwarz

**Affiliations:** ^1^ Department of Surgery, Indiana University School of Medicine, South Bend, IN 46617, USA; ^2^ Department of Chemistry and Biochemistry, University of Notre Dame, Notre Dame, IN 46617, USA; ^3^ Department of Pediatrics, Indiana University School of Medicine, South Bend, IN 46617, USA; ^4^ Harper Cancer Research Institute, University of Notre Dame, Notre Dame, IN 46617, USA; ^5^ Goshen Center for Cancer Care, Goshen, IN 46526, USA

**Keywords:** pancreatic cancer, nab-paclitaxel, trametinib, MEK inhibitor, combination therapy

## Abstract

Nab-paclitaxel (NPT) combination with gemcitabine (Gem) represents the standard chemotherapy for pancreatic ductal adenocarcinoma (PDAC). Genetic alterations of the RAS/RAF/MEK/ERK (MAPK) signaling pathway yielding constitutive activation of the ERK cascade have been implicated as drivers of PDAC. Inhibition of downstream targets in the RAS-MAPK cascade such as MEK remains a promising therapeutic strategy. The efficacy of trametinib (Tra), a small molecule inhibitor of MEK1/2 kinase activity, in combination with nab-paclitaxel-based chemotherapy was evaluated in preclinical models of PDAC. The addition of trametinib to chemotherapy regimens showed a trend for an additive effect on tumor growth inhibition in subcutaneous AsPC-1 and Panc-1 PDAC xenografts. In a peritoneal dissemination model, median animal survival compared to controls (20 days) was increased after therapy with NPT (33 days, a 65% increase), Tra (31 days, a 55% increase), NPT+Tra (37 days, a 85% increase), NPT+Gem (39 days, a 95% increase) and NPT+Gem+Tra (49 days, a 145% increase). Effects of therapy on intratumoral proliferation and apoptosis corresponded with tumor growth inhibition. Trametinib effects were specifically accompanied by a decrease in phospho-ERK and an increase in cleaved caspase-3 and cleaved PARP-1 proteins. These findings suggest that the effects of nab-paclitaxel-based chemotherapy can be enhanced through specific inhibition of MEK1/2 kinase activity, and supports the clinical application of trametinib in combination with standard nab-paclitaxel-based chemotherapy in PDAC patients.

## INTRODUCTION

Pancreatic ductal adenocarcinoma (PDAC) is among the most lethal malignancies in the US [1]. PDAC is currently the third most frequent cause of cancer deaths in the US and is expected to be the second deadliest cancer by 2030 [1, 2]. The 5-year survival rate in PDAC patients remains about 6% and the poor prognosis is attributed to several factors including late-stage diagnosis, an aggressive progression of the disease and high resistance to conventional therapies. Surgery remains the best option for the long-term survival of PDAC patients, however, only about 20% of patients are suitable for this procedure [3]. Furthermore, post-operative recurrence remains very common, and even after complete resection, the 5-year survival is only around 25% [1]. Therefore, improving systemic treatment strategies is highly desirable for PDAC patients. Gemcitabine (Gem) remained a standard therapy for PDAC for more than 16-years despite only a minimal clinical benefit [4]. FOLFIRINOX is a combination chemotherapy regimen that increased the median survival of unresectable PDAC patients to approximately 11 months. However, this regimen has a high toxicity potential, limiting its use to only patients with good performance status [5]. Nab-paclitaxel (NPT) is a next-generation taxane that has demonstrated significant antitumor efficacy in several solid tumors. Nab-paclitaxel is an approved treatment for breast cancer, NSCLC and PDAC [6]. Nab-paclitaxel combined with gemcitabine has recently become the standard treatment for unresectable PDAC after demonstrating a 1.8 months improvement in patient survival compared to gemcitabine monotherapy [7]. Due to the limited clinical efficacy of current cytotoxic chemotherapy regimens for PDAC patients, novel therapeutic approaches are urgently needed to further improve patient survival.

Activation of ERK signaling due to mutations in the RAS/RAF/MEK/ERK (MAPK) pathway has been implicated in several cancers [8]. In PDAC, activating *KRAS* mutations occur at a frequency of 90% [9], rendering this a potential therapeutic target of great interest. Developing drugs that directly target mutant KRAS protein remains challenging due to target specificity issues [10]. Therefore, alternative strategies focus on inhibition of downstream targets of the RAS-MAPK cascade. MEK is one such protein kinase located downstream of RAS/RAF, making it an attractive target for cancer therapy [11]. Trametinib (Tra, Supplementary Figure 1), a selective and reversible small molecule inhibitor of MEK1/2 kinase activity, has demonstrated antitumor efficacy in preclinical studies of several tumor types, with the largest effect in tumors harboring mutant BRAF or Ras [12]. Furthermore, trametinib is an FDA-approved treatment for V600E-mutant metastatic melanoma patients as a single agent or in combination with dabrafenib [13, 14]. In a preclinical study with patient-derived xenografts of PDAC, trametinib showed significant antitumor effects [15]. In addition, the first clinical study of trametinib monotherapy in PDAC demonstrated potential activity [16]. In a recent phase II clinical trial in PDAC patients, trametinib combination with gemcitabine demonstrated a 1.7 months increase in median overall survival (OS) compared with the gemcitabine alone. However, this difference in OS was evaluated as non-significant as the observed separation was not durable with a hazard ratio of 0.98 [17]. Furthermore, in a phase II clinical study in *KRAS* mutant NSCLC patients, selumetinib, another MEK inhibitor, plus docetaxel showed significant improvements in response rate and progression-free survival (PFS) [18], indicating differences in the synergy of MEK inhibitors with gemcitabine compared with taxanes. In this study, we report the antitumor efficacy of trametinib in combination with nab-paclitaxel-based chemotherapy regimens in preclinical models of pancreatic cancer.

## RESULTS

### Nab-paclitaxel-based chemotherapy regimens and trametinib reduce tumor growth

**Figure 1:**
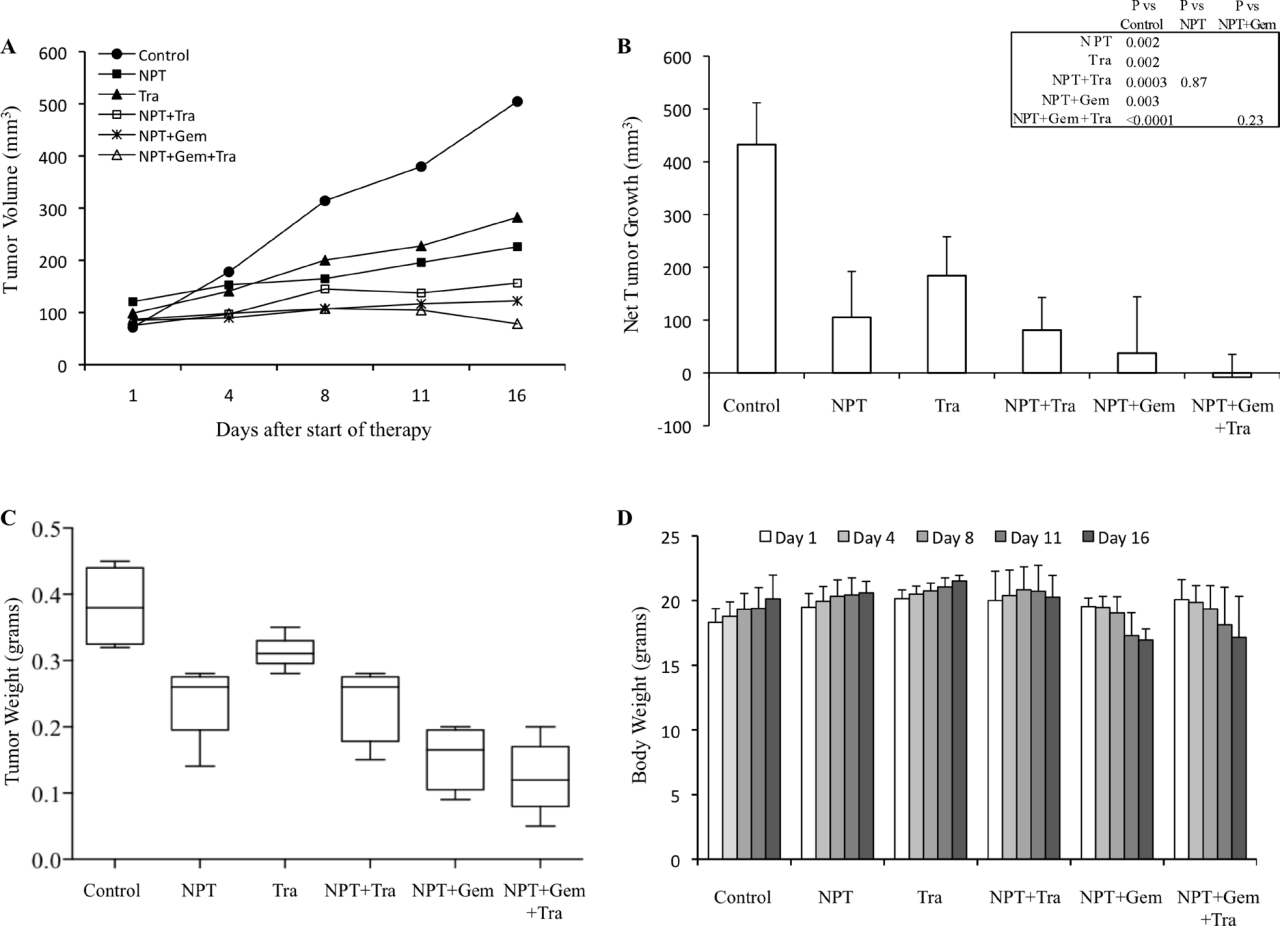
Antitumor activity of nab-paclitaxel-based chemotherapy regimens and trametinib in AsPC-1 tumor xenografts AsPC-1 cells were subcutaneously injected into nude mice and treated with nab-paclitaxel-based chemotherapy regimens and trametinib for 2 weeks. (**A**) Tumor size as measured twice-weekly using calipers. (**B**) Net tumor growth, calculated by subtracting tumor volume on the first treatment day from that on the final day. (**C**) Mean tumor weight was calculated from final day tumor weights in each group, presented as a Box plot. (**D**) Mouse body weight was measured twice a week and presented as bar chart for the 2-week therapy period. Data are representative of mean values ± standard deviation from 5 mice per group.

**Figure 2:**
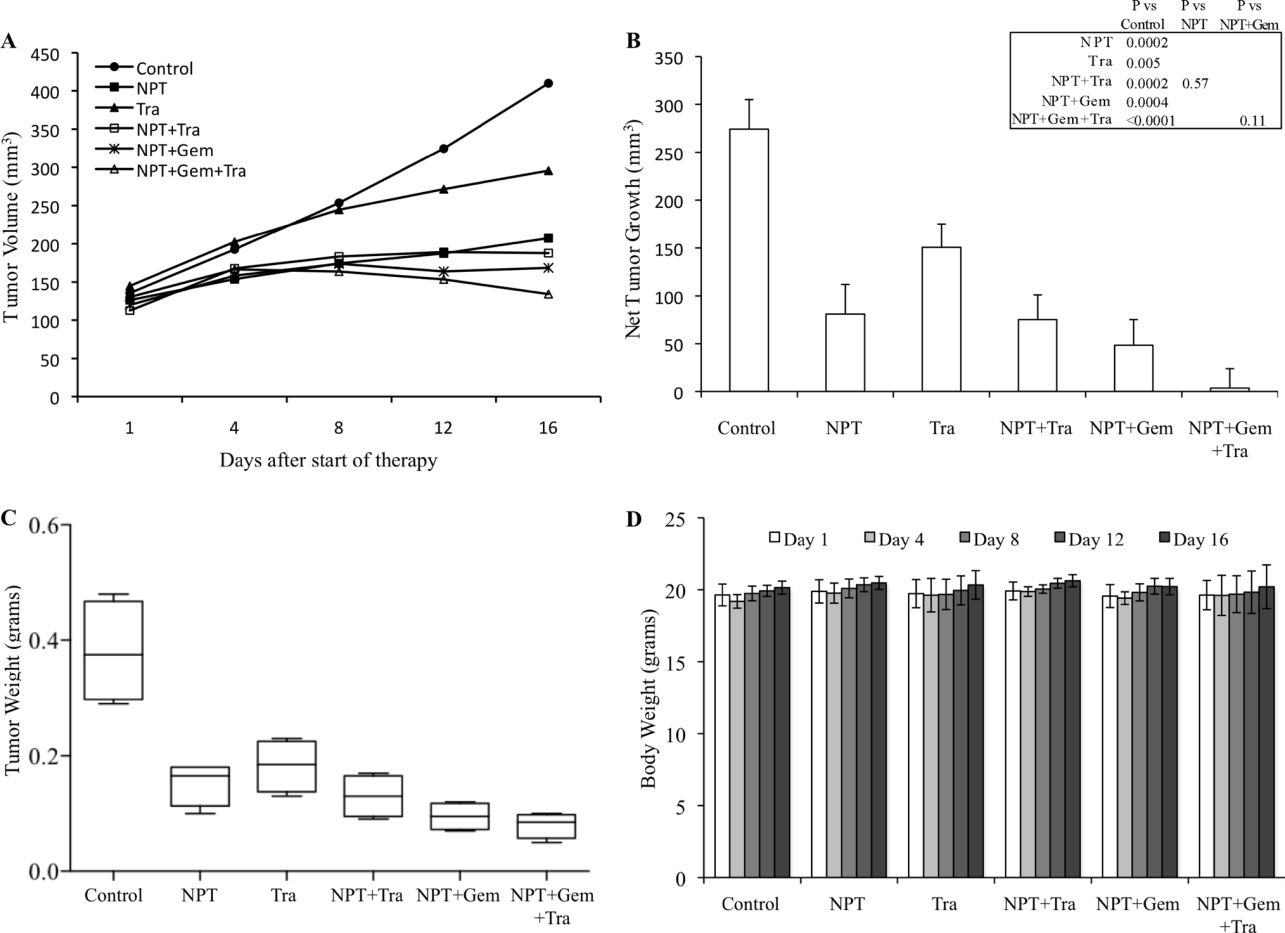
Antitumor activity of nab-paclitaxel-based chemotherapy regimens and trametinib in Panc-1 tumor xenografts Panc-1 cells were subcutaneously injected in nude mice and treated with nab-paclitaxel-based chemotherapy regimens and trametinib for 2 weeks. (**A**) Tumor size as measured twice-weekly using calipers. (**B**) Net tumor growth, calculated by subtracting tumor volume on the first treatment day from that on the final day. (**C**) Mean tumor weight was calculated from final day tumor weights in each group and is presented as a Box plot. (**D**) Mouse body weight was measured twice a week and presented as bar chart for the 2-week therapy period. Data are representative of mean values ± standard deviation from 5 mice per group.

In an AsPC-1 subcutaneous xenograft model, nab-paclitaxel alone, nab-paclitaxel plus gemcitabine and trametinib alone caused an inhibition in tumor growth while trametinib combination with chemotherapy regimens had a trend for additive effects (Figure 1A). Net tumor growth in different groups after 2-weeks of therapy was 432.6 mm^3^ in controls, 105.3 mm^3^ after NPT, 184 mm^3^ after Tra, 81 mm^3^ after NPT+Tra, 37.3 mm^3^ after NPT+Gem and –8.1 mm^3^ (tumor regression) after NPT+Gem+Tra (Figure 1B). Tumor weight at the completion of the experiment in different groups was 0.38 g in controls, 0.23 g in NPT, 0.31 g in Tra, 0.23 g in NPT+Tra, 0.15 g in NPT+Gem and 0.11 g in NPT+Gem+Tra (Figure 1C). In another Panc-1 subcutaneous xenograft model, nab-paclitaxel, nab-paclitaxel plus gemcitabine and trametinib therapy also caused an inhibition in tumor growth rates with a trend for additive effects in combination groups (Figure 2A). Net tumor growth in different groups was 274.1 mm^3^ in controls, 80.8 mm^3^ after NPT, 150.6 mm^3^ after Tra, 75.1 mm^3^ after NPT+Tra, 48.4 mm^3^ after NPT+Gem and 3.8 mm^3^ after NPT+Gem+Tra (Figure 2B). Tumor weight at the completion of the experiment in different groups was: 0.37 g in controls, 0.15 g in NPT, 0.18 g in Tra, 0.13 g in NPT+Tra, 0.09 g in NPT+Gem and 0.077 g in NPT+Gem+Tra (Figure 2C). Also, in these two subcutaneous xenograft experiments, no disenable therapy-related toxicity was observed during the therapy period and there was no significant change in the body weight of mice in all groups (Figures 1D, 2D).

### Nab-paclitaxel-based chemotherapy regimens and trametinib increase animal survival

**Figure 3:**
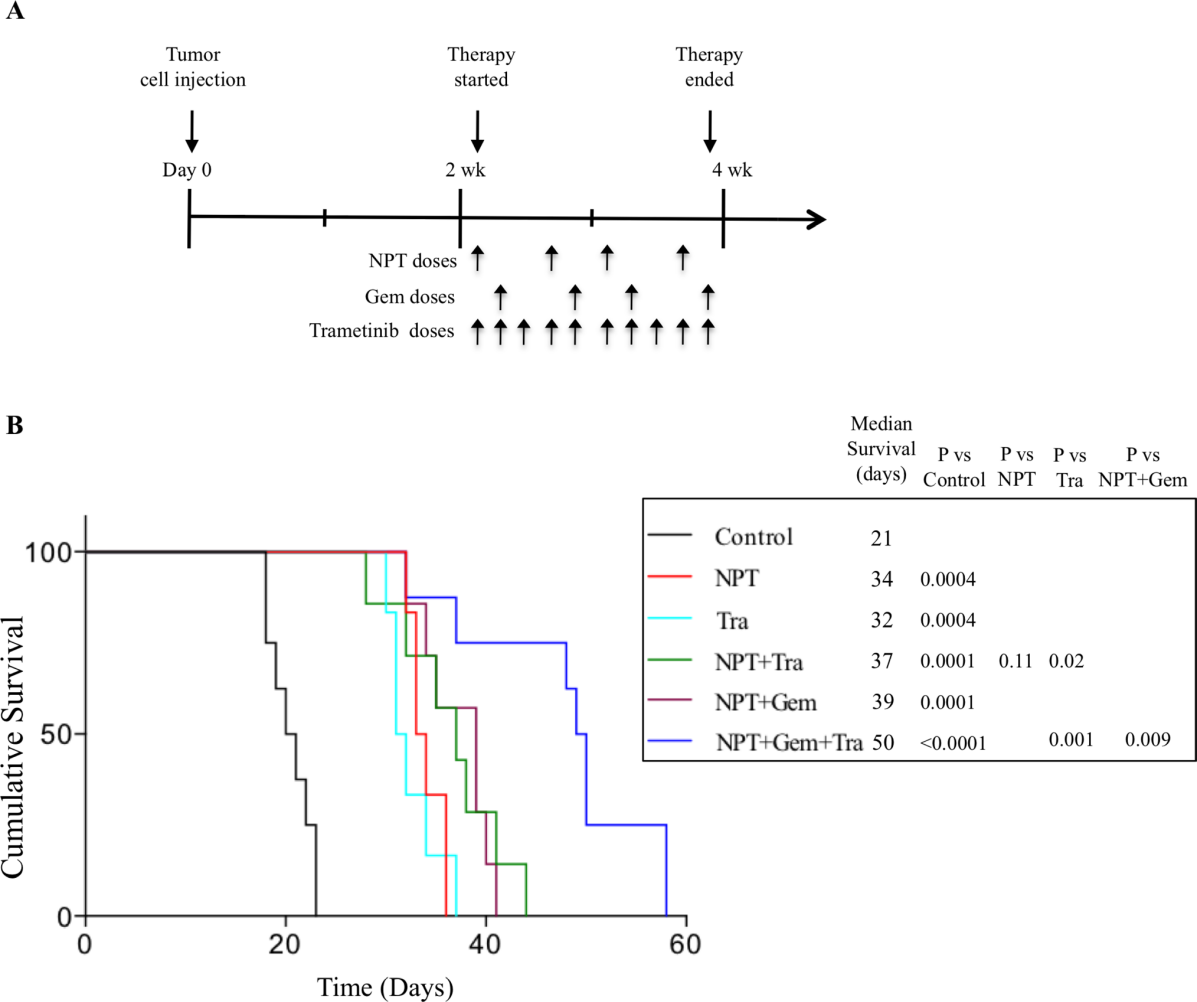
Prolongation of animal survival by nab-paclitaxel-based chemotherapy regimens and trametinib (**A**) Schematic representation of experimental procedure. AsPC-1 cells (0.75 × 10^6^) were injected intraperitoneally in NOD/SCID mice and treatment was started after 2 weeks with nab-paclitaxel, gemcitabine and trametinib for 2 weeks. (**B**) The curve represents the animal survival time from the beginning of therapy. Statistical group differences in survival time were calculated using logrank testing.

In an AsPC-1 pancreatic cancer peritoneal dissemination model, animal survival in different groups, compared with controls (20 days) was increased after therapy with NPT (33 days, a 65% increase), Tra (31 days, a 55% increase), NPT+Tra (37 days, a 85% increase), NPT+Gem (39 days, a 95% increase) and NPT+Gem+Tra (49 days, a 145% increase) (Figure 3A, 3B). No significant change in the body weight of mice was observed during 2-week therapy period, indicating that there was no discernable therapy-associated toxicity in all therapy groups.

### Nab-paclitaxel-based chemotherapy and trametinib: tumor cell proliferation and apoptosis

The antitumor mechanisms of nab-paclitaxel-based chemotherapy regimens and trametinib were investigated by IHC analysis of subcutaneous tumor tissues.

**Figure 4:**
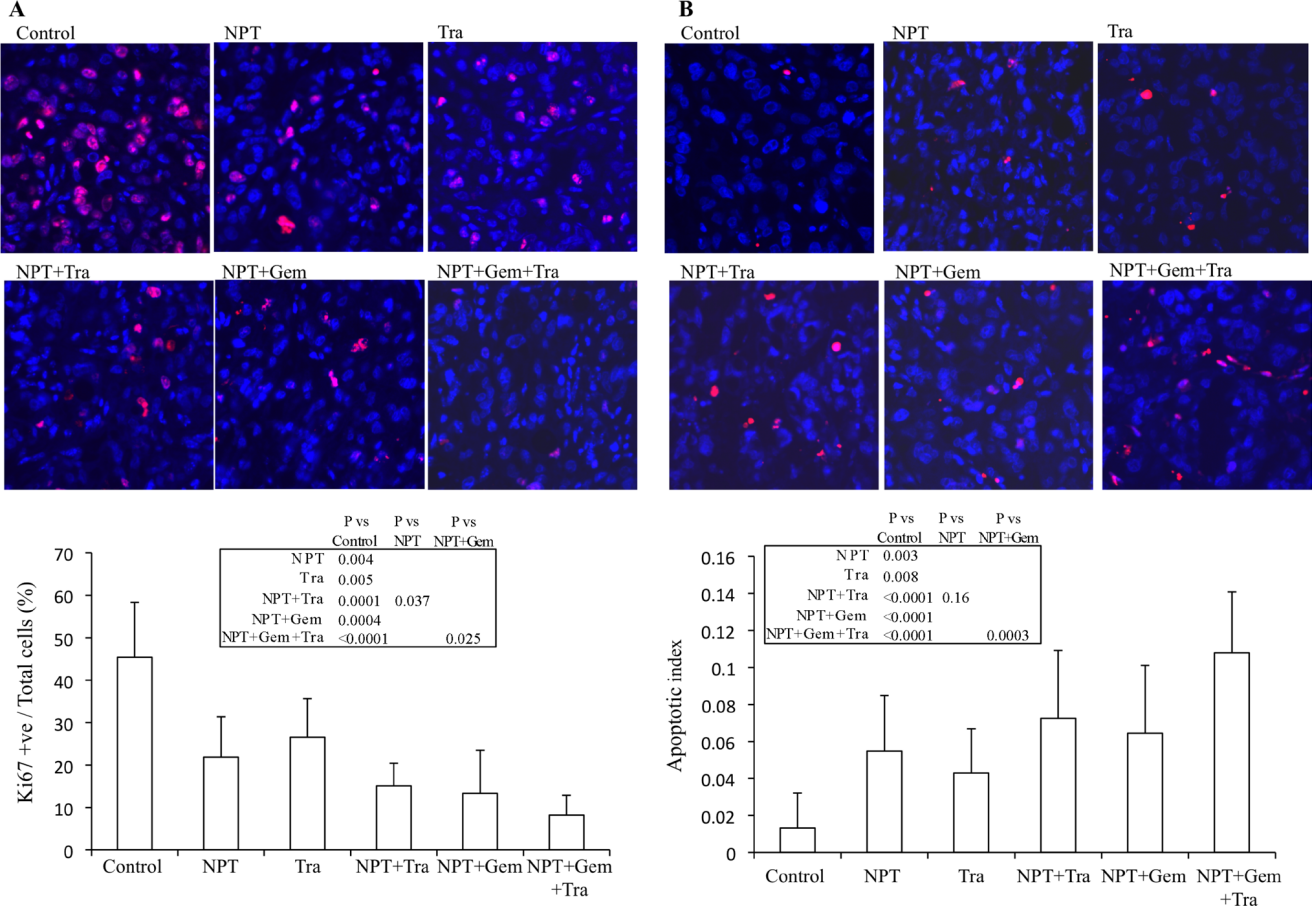
Mechanisms of antitumor activity of nab-paclitaxel-based chemotherapy regimens and trametinib Nude mice were subcutaneously injected with AsPC-1 cells and treated with nab-paclitaxel, gemcitabine and trametinib for 2 weeks. (**A**) Intratumoral proliferation was measured by immunostaining tissue sections for Ki67 nuclear antigen. Ki67-positive cells were counted in five different high power fields. (**B**) Intratumoral apoptosis was measured by staining tumor tissue section with TUNEL procedure. TUNEL-positive apoptotic cells were counted in five different high power fields. For both immunostaining experiments, slides were photographed under a fluorescent microscope and the data are expressed as the mean ± standard deviation.

Ki67 staining demonstrated that nab-paclitaxel, nab-paclitaxel plus gemcitabine and trametinib decreased tumor cell proliferation and combination therapy groups demonstrated a trend for an additive effect. Intratumoral proliferative index, the percentage of Ki67-positive cells over the total number of cells per high-power field, in different therapy groups was: controls (45.4), NPT (21.8), Tra (26.6), NPT+Tra (15.1), NPT+Gem (13.3) and NPT+Gem+Tra (8.2) (Figure 4A).

TUNEL assay detected that nab-paclitaxel and trametinib monotherapies increased tumor cell apoptosis and the combination therapy groups demonstrated a trend for an additive effect. The apoptotic index readings, by therapy group, were: controls (0.013), NPT (0.055), Tra (0.043), NPT+Tra (0.072), NPT+Gem (0.064) and NPT+Gem+Tra (0.108) (Figure 4B).

**Figure 5:**
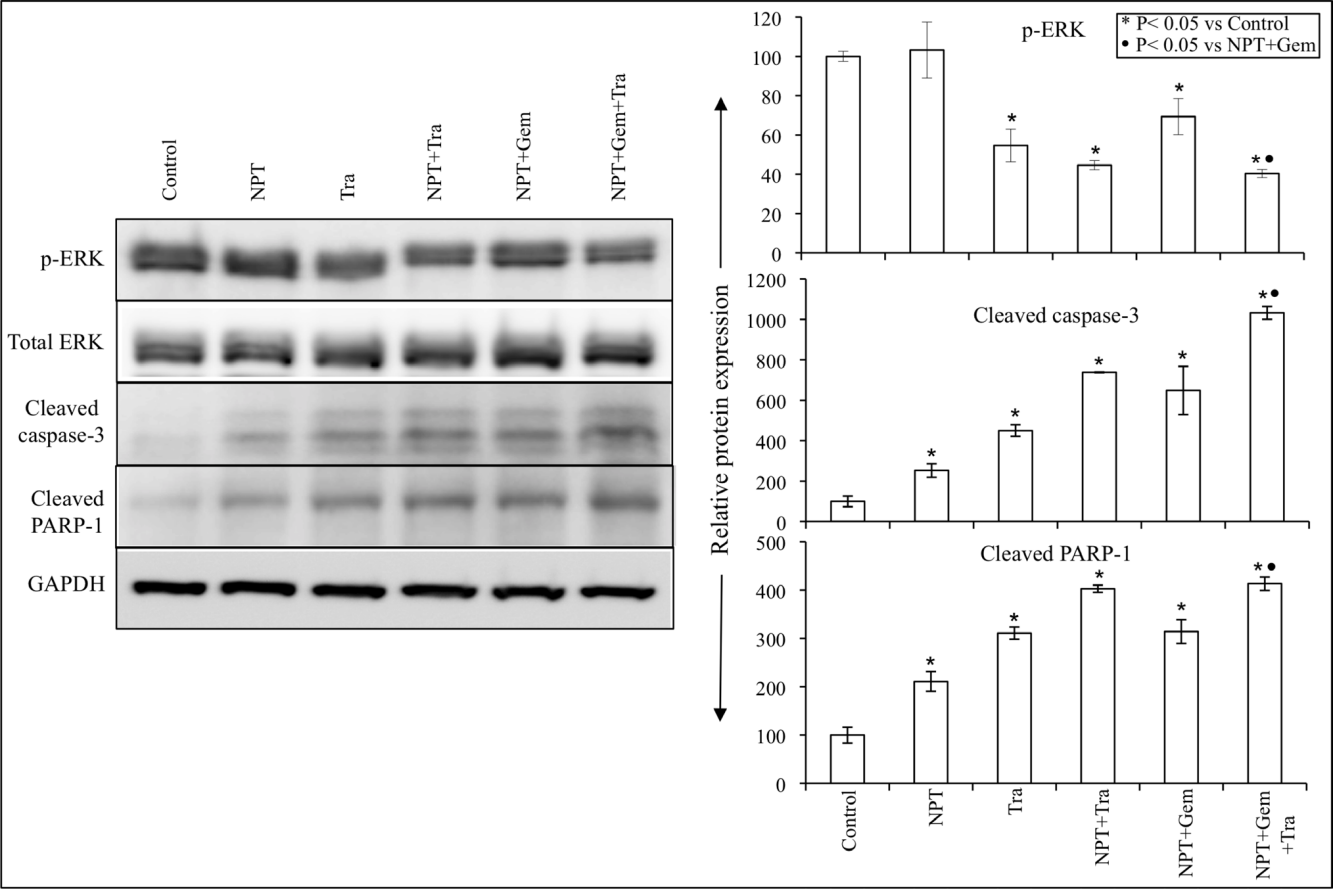
Nab-paclitaxel-based chemotherapy regimens and trametinib effects on their molecular targets *in vivo* Tumor lysates were prepared from tumor tissues obtained from AsPC-1 tumor-bearing mice and were analyzed by immunoblotting. Data are representative of pooled lysates obtained from tumor sections of at least 5 mice in each therapy group. The intensity of bands was quantitated by densitometry and is represented in the bar graph after normalizing values with corresponding total protein expression or GAPDH expression.

Further investigation of the tumor growth inhibitory action of nab-paclitaxel-based chemotherapy regimens and trametinib exhibited that trametinib significantly decreased ERK phosphorylation and concomitantly increased the expression of apoptosis-associated proteins cleaved caspase-3 and cleaved PARP-1 (Figure 5).

### Nab-paclitaxel-based chemotherapy and trametinib: *In vitro* cell viability and cellular targets

**Figure 6:**
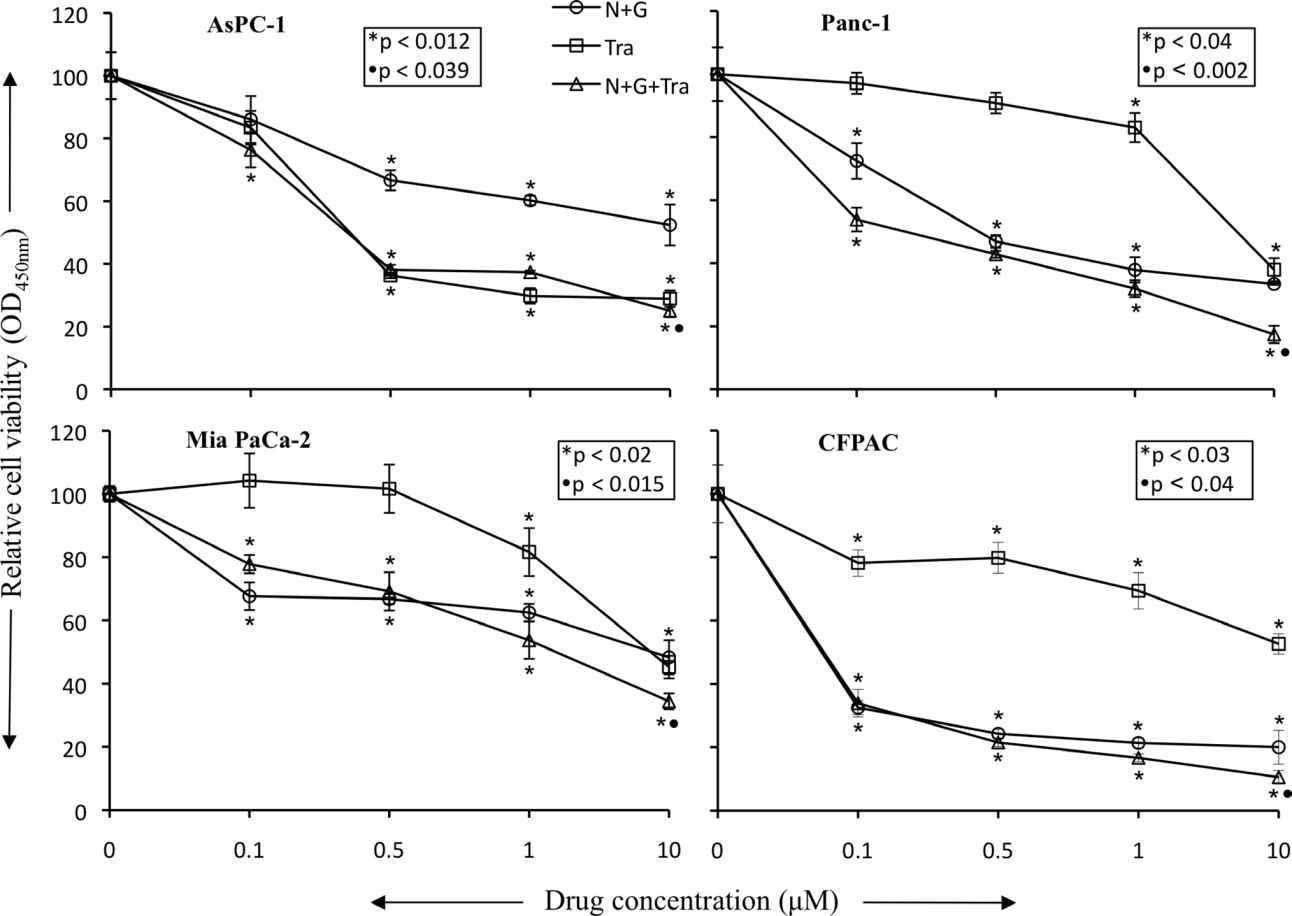
Nab-paclitaxel plus gemcitabine and trametinib inhibit *in vitro* PDAC cell proliferation PDAC cells (AsPC-1, Panc-1, Mia PaCa-2, CFPAC) were plated on 96-well plates and treated with 100 nM to 10 μM concentrations of nab-paclitaxel, gemcitabine and trametinib. After 72 hours, 10 μl WST-1 reagent was added in each well and incubated for 2 additional hours. The absorbance at 450 nm was measured using a microplate reader. The resulting number of viable cells was calculated by measuring absorbance of color produced in each well. Data are the mean ± SD of triplicate determinations. The *p* values represent significance of the difference between the control and treated groups.

**Figure 7:**
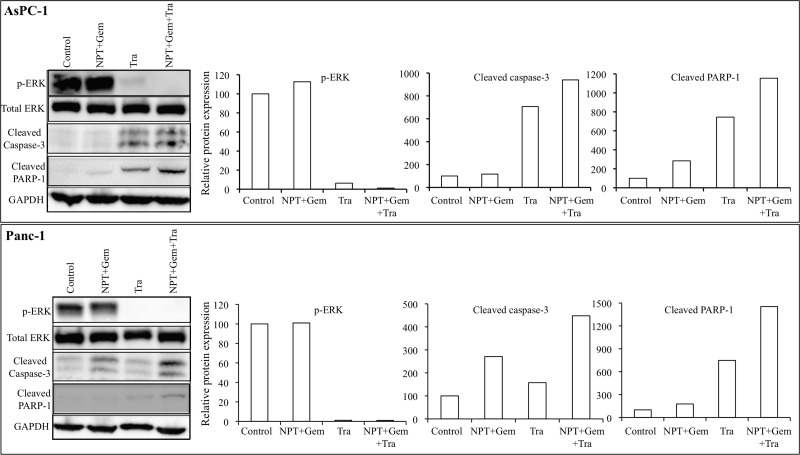
Nab-paclitaxel-based chemotherapy regimens and trametinib effects on their molecular targets *in vitro* A sub-confluent monolayer of human PDAC cells AsPC-1 and Panc-1 was treated with nab-paclitaxel (10 μM), gemcitabine (10 μM) and trametinib (10 μM), either alone or in combination for 16 hours. Total cell extracts were analyzed by immunoblotting. The intensity of bands was quantitated by densitometry and is represented in the bar graph after normalizing values against corresponding total protein expression or GAPDH expression. Data are representative of two independent experiments with similar results.

*In vitro* cell viability examination of human PDAC epithelial cells with different mutations [19], indicated that both, nab-paclitaxel plus gemcitabine and trametinib, suppressed cell proliferation. In the combination therapy groups, at high dose (10 μM), a statistically significant additive response on cell proliferation inhibition was observed in N+G+Tra compared with N+G or Tra groups for all the four cell lines tested (Figure 6). Nab-paclitaxel plus gemcitabine treatment dose-dependently inhibited cell proliferation, and at 10 μM concentration of each drug inhibition in proliferation was 47.6% (AsPC-1), 66.6% (Panc-1), 51.7% (Mia PaCa-2) and 80% (CFPAC) (Figure 6). Addition of trametinib (10 μM) to the nab-paclitaxel plus gemcitabine treatment group caused an additive effect and inhibition in cell proliferation in this combination group was 75% (AsPC-1), 82.6% (Panc-1), 65.6% (Mia PaCa-2) and 89.4% (CFPAC) (Figure 6). Immunoblot analysis determined that trametinib blocked the expression of phospho-ERK and concomitantly increased the expression of cleaved caspase-3 and cleaved PARP-1 proteins (Figure 7).

## DISCUSSION

Pancreatic cancer is frequently refractory to cytotoxic and targeted therapies, mainly due to the marked genetic heterogeneity and resultant complexity in molecular signaling [20]. Specifically, PDACs exhibit a high frequency of activating mutations in the *KRAS* oncogene, inactivating mutations in tumor suppressor genes *p16/CDKN2A* and *TP53* and inactivation of the *SMAD4* gene [21–23]. Activating mutation in the *KRAS* oncogene is a hallmark of PDAC as it is crucial for PDAC growth and progression and occurs in approximately 95% of patients [24]. Further, the importance of the *KRAS* oncogene in PDAC development has been demonstrated in genetically engineered mouse models [25, 26]. Therefore, investigating strategies for effective inhibition of KRAS signaling is critically important in pancreatic cancer research [25, 27]. Small molecule inhibitors of downstream targets of the RAS-RAF-MEK-ERK pathway are in clinical development and have shown promising efficacy in RAS/RAF-driven tumors [28, 29].

Trametinib has been shown to prevent RAS-dependent MEK phosphorylation and cause prolonged inhibition of ERK signaling [12]. Trametinib is highly selective for their targets, which may lead to fewer off-target effects [30]. In PDAC, trametinib augmented combined treatment with EGFR and HER2 inhibitors in patient-derived xenograft models [15, 31]. Early clinical studies in PDAC with trametinib monotherapy or its combination with gemcitabine showed promising results [16, 32]. Although a phase II clinical trial in untreated metastatic PDAC patients could not demonstrate significant clinical efficacy of the combination of gemcitabine and trametinib [17], another study in NSCLC with selumetinib as a MEK inhibitor demonstrated significant clinical benefits in combination with docetaxel, indicating the possibility of some synergy potential of MEK inhibitors with taxanes compared with gemcitabine [18]. Therefore, we explored the antitumor benefits of combining trametinib with the current standard of care chemotherapy regimen containing both the next-generation taxane nab-paclitaxel and gemcitabine in preclinical models of pancreatic cancer.

We detected a significant expression of phospho-MEK and phospho-ERK (Supplementary Figure 2) in all seven PDAC cells tested, confirming the prevalence of the MAPK signaling cascade in PDAC cellular activation and progression. In subcutaneous xenografts of two aggressive *KRAS*-mutant cell lines, AsPC-1 and Panc-1, we determined that trametinib not only inhibited tumor growth as a monotherapy, but it also demonstrated a trend for an additive effect in combination with chemotherapy regimens, although it did not reach statistical significance (Figures 1, 2). The trend for additive effect was more pronounced in the NPT+Gem group compared to the NPT group. Tumor cell proliferation and apoptosis rate evaluated in tumor tissue correlated with the tumor growth experiment results, supporting a trend for an additive effect on decreased proliferation and increased apoptosis by addition of trametinib (Figure 4). Furthermore, the addition of trametinib to chemotherapy regimens caused an obvious and statistically significant increase in animal survival in our peritoneal dissemination model (Figure 3). Smaller additive effects of trametinib in the subcutaneous model suggest the possibility of the local tumor microenvironment factors making MEK inhibition less relevant to PDAC growth compared with the more clinically relevant peritoneal dissemination model where MEK inhibition caused a significant improvement in the chemotherapy response. Target protein determination in protein lysates from subcutaneous xenografts showed that trametinib significantly decreased phosphorylated ERK and increased the expression of apoptosis-associated proteins, suggesting that the trametinib therapy is sufficiently affecting its target within the local tumor setting. Early MEK inhibitors such as PD0325901, selumetinib and cobimetinib blocked ERK activation, but concomitant interference with ERK-dependent negative feedback resulted in the accumulation of phospho-MEK and rebound in ERK signaling [33, 34]. Newer MEK inhibitors including trametinib cause minimal to no increase in phospho-MEK or recovery in ERK activity, resulting in lasting inhibition of MAPK pathway and superior antitumor activity [34].

Previous studies in our laboratory have employed an established peritoneal dissemination model of PDAC to study animal survival using *KRAS*-mutant AsPC-1 cells that resembles the human disease in its aggressiveness and metastatic progression [35, 36]. With this approach, we observed that animal survival was improved by trametinib with and without nab-paclitaxel or nab-paclitaxel plus gemcitabine. More importantly, trametinib augmented nab-paclitaxel-based chemotherapy effects for the greatest improvement in animal survival. These results are consistent with a recent study demonstrating that trametinib in combination with gemcitabine had enhanced antitumor efficacy in a patient-derived xenograft model of PDAC in inhibiting growth rate of liver metastases and increasing PFS [37]. This observation is through our data extended to nab-paclitaxel, which appears to be specifically suited for *in vivo* therapy benefits when combined with trametinib. Although the appropriate sequence for the administration of MEK inhibitor and chemotherapy agents has been shown to impact clinical outcomes [17, 38], we have not evaluated the effects of varied dosing schedule of these drugs. In this study, nab-paclitaxel effects on enhanced tissue distribution and tumor penetration of drugs might be partially responsible for enhanced antitumor response in combination therapy groups as nab-paclitaxel therapy was started together with trametinib and a day before gemcitabine [39].

*In vitro* cell viability analysis revealed that trametinib inhibited proliferation of *KRAS*-mutant PDAC cells while the combination of trametinib with nab-paclitaxel plus gemcitabine had additive effects on inhibition in cell proliferation at high dose level (10 μM). Among these cells, AsPC-1 cells were most sensitive to trametinib and a significant decrease in cell viability was observed at all dose levels, and a very little additive benefit on cell viability inhibition was observed by addition of trametinib to chemotherapy. Other PDAC cells tested showed a small effect on cell viability at low dose levels and the effect was significant at high dose levels. The differential sensitivity of pancreatic cancer cells to MEK inhibition can be attributed to the KRAS mutational subtype, copy number and the presence of PIK3CA co-mutation [40, 41]. In a phase 1 study, the maximum plasma concentration of trametinib was 62.8 ng/ml (∼0.1 μM) [16]. Similar to the target protein evaluation in tumor lysates, protein lysates from AsPC-1 and Panc-1 cells demonstrated a dramatic decrease in ERK phosphorylation and an increase in apoptosis-related proteins by trametinib treatment. These findings further indicate that the antitumor response of trametinib is linked to blocking ERK induced molecular signaling changes that are accountable for tumor cell proliferation and survival.

PDAC progression is multifactorial involving increased tumor cell proliferation, differentiation, migration, angiogenesis, epithelial-to-mesenchymal transition, desmoplasia and immune system evasion, and most of these mechanisms have been shown to be affected by or regulated through the RAS-RAF-MEK-ERK signaling pathway [42, 43]. We have previously demonstrated that nab-paclitaxel-based chemotherapy has antitumor responses based on its antiproliferative, antistromal and proapoptotic effects [44]. Therefore, the possible molecular mechanisms for the improvement in antitumor responses of nab-paclitaxel-based chemotherapy regimen by trametinib may likely be caused by not only augmenting antiproliferative and proapoptotic activities but also by inhibiting other tumorigenic pathways including angiogenesis [45].

Based on the fact that the RAS-RAF-MEK-ERK pathway is one of the most frequently dysregulated pathways in PDAC, MEK inhibitors have shown promising antitumor activity, either alone or in combination with other anticancer drugs [46–48]. Trametinib is a new generation MEK1/2 inhibitor, which is highly selective for these targets. Compared with other MEK inhibitors, trametinib has several advantages including a favorable pharmacokinetic profile, long half-life and manageable toxicity [16, 49]. In the present study, no discernible treatment-associated toxicity was observed during a 2-week therapy period, however, toxicity associated with long-term therapy of trametinib in combination with nab-paclitaxel-based chemotherapy regimens remains to be elucidated. In conclusion, our study indicates that trametinib, a MEK1/2 inhibitor blocking *KRAS* activating mutation induced RAF-MEK-ERK tumorigenic signaling, is an effective mechanism-specific antitumor agent, and it can improve nab-paclitaxel-based chemotherapy effects in PDAC. These results strongly support the rationale of blocking downstream targets of *KRAS*-mutation driven signaling and indicate the potential of trametinib as a targeting agent in combination with nab-paclitaxel-based chemotherapy regimens for clinical PDAC therapy.

## MATERIALS AND METHODS

### Cell culture and reagents

Human PDAC cell lines (AsPC-1, BxPC-3, CFPAC, Mia PaCa-2 and Panc-1) were purchased from the American Type Culture Collection (ATCC, Rockville, MD). Cell lines were tested and authenticated by ATCC. The most common mutations in all the cell lines used in this study are as follows: AsPC-1 (KRAS, p53, p16), Panc-1 (KRAS, p53, p16), Mia PaCa-2 (KRAS, p53, p16) and CFPAC (KRAS, p53, SMAD4) [19]. Cells were initially grown and multiple aliquots were cryopreserved. All the cell lines were used within 6 months after reexpansion in culture. Cells were cultured in DMEM or RPMI 1640 medium (Sigma Chemical Co. St. Louis, MO) containing 10% FBS and maintained at 37°C in a humidified incubator with 5% CO_2_ and 95% air. Nab-paclitaxel was obtained from Celgene Corporation (Summit, NJ). Gemcitabine and trametinib were purchased from LC labs (Woburn, MA). The cell proliferation reagent WST-1 was purchased from Roche Diagnostic Corporation (Indianapolis, IN).

### Cell viability assay

Cell viability was evaluated by the colorimetric WST-1 assay as previously described [50]. Briefly, PDAC cells (4,000 to 5,000 cells per well) were plated in a 96-well plate in regular cell growth medium containing 10% FBS. After 16 hours the medium was replaced with low serum medium containing 2% FBS and the cells were treated with 100 nM to 10 μM concentrations of nab-paclitaxel, gemcitabine and trametinib. After 72 hours, 10 μl WST-1 reagent was added in each well followed by additional incubation for 2 hours. The absorbance was measured at 450 nm using a microplate reader.

### Western blot analysis

Protein lysates were prepared by treating sub-confluent cells with nab-paclitaxel (10 μM), gemcitabine (10 μM) and trametinib (10 μM), and lysed after 16 hours for Western blotting as previously described [50]. Protein lysates of subcutaneous tumors were prepared by snap freezing tumor tissues in liquid nitrogen and stored at –80°C. These tumor tissues were suspended in lysis buffer and homogenized using the Bullet Blender Homogenizer (Next Generation, Averill Park, NY), and extracts were sonicated on ice. Proteins in supernatants were separated by SDS-PAGE and transferred to PVDF membranes (Bio-Rad, Hercules, CA). The membranes were incubated overnight at 4°C with the following antibodies: total ERK1/2, phospho-ERK1/2 (Thr202/Tyr204), cleaved caspase-3, cleaved PARP-1 and GAPDH (Cell Signaling Technology, Beverly, MA). The membranes were then incubated with the corresponding HRP-conjugated secondary antibodies (Pierce Biotechnologies, Santa Cruz, CA) for 1 to 2 hour. Protein bands were visualized using the enhanced chemiluminescence reagent (SignalFire, Cell Signaling) with an Image360 system and quantitated by densitometry.

### Animal experiments

All animals were housed in a pathogen-free facility with access to food and water *ad libitum*. Animal experiments were performed in accordance with the Institutional Animal Care and Use Committee (IACUC) at the Indiana University School of Medicine (South Bend, IN). Female nonobese diabetic/severe combined immunodeficient (NOD/SCID) mice (4 to 6 weeks old) were subcutaneously injected with AsPC-1 cells (7.5 × 10^5^) or Panc-1 cells (10 × 10^6^) as previously described [44]. Two weeks after tumor cell injection, all mice had a measurable tumor. Mice were then randomized (*n* = 4 to 6 per group) to receive PBS (control), nab-paclitaxel (5 mg/kg, twice a week), gemcitabine (50 mg/kg, twice a week) and trametinib (1 mg/kg, 5 times a week) via intraperitoneal injection for next 2 weeks. The tumor size was measured twice weekly, and tumor volume (V) was calculated using the formula V = ½ (Length × Width^2^). Net tumor growth was calculated by subtracting tumor volume on the first therapy day from that on the last day. Mice were euthanized after completion of treatment, tumors were dissected and processed for histological, immunohistochemical and Western blot analysis.

Animal survival studies were performed using female NOD/SCID mice (4–6 weeks of age) as previously described [51]. Briefly, the mice were injected intraperitoneally with AsPC-1 (7.5 × 10^5^) cells and two weeks after tumor cell injection, mice were randomized (*n* = 6 to 8 per group) to receive PBS (control), nab-paclitaxel (5 mg/kg, twice a week), gemcitabine (50 mg/kg, twice a week) and trametinib (1 mg/kg, 5 times a week) via IP injection for two weeks. Animals were euthanized when moribund according to predefined criteria [52, 53]. Animal survival was evaluated from the first day of treatment until death.

### Immunohistochemistry and immunofluorescence

Standard immunohistochemistry protocol was followed to stain the tumor tissue sections, as previously described [54]. Briefly, tumor tissue samples were fixed in 4% paraformaldehyde, embedded in paraffin, tissue sections were cut (5 μm), deparaffinized and rehydrated. The tissue sections were incubated with 1:200 dilution of Ki67 antibody (ab15580, Abcam, Cambridge, MA) followed by incubation with 1:200 dilution of anti-rabbit-Cy3 secondary antibody (Jackson ImmunoResearch Laboratories, West Grove, PA). Slides were mounted using a mounting solution containing 4′,6-diamidino-2-phenylindole (DAPI) (Invitrogen, Carlsbad, CA). The intratumoral proliferative index was determined by calculating the Ki67-positive cells from five different high-power fields (HPF) in a blinded manner in each group. Intratumoral apoptotic activity was evaluated by staining tissue sections with “Apoptag Apoptosis Detection Kit” according to the manufacturer's (Millipore, Temecula, CA) instructions. The apoptotic index was calculated by dividing the number of TUNEL-positive cells by the total number of cells per HPF in a blinded manner in each group. Fluorescence microscopy was used to detect fluorescent signals using the IX81 Olympus microscope equipped with a Hamamatsu Orca digital camera (Hamamatsu Corporation, Bridgewater, NJ) and a disk-scanning unit (DSU) spinning disk confocal system using Slidebook software (Intelligent Imaging Innovations, Philadelphia, PA).

### Statistical analysis

Statistical analysis for *in vivo* tumor growth studies was performed by one-way ANOVA for multiple group comparisons and Student's *t*-test for the individual group comparisons. Survival study statistics were performed using logrank group comparison (GraphPad Prism 6.0). *P* values less than 0.05 were considered statistically significant. *In vitro* cell proliferation data are expressed as the mean ± standard deviation. Statistical significance was analyzed by the two-tailed Student's *t*-test using GraphPad Prism 6.0 Software (GraphPad Software, San Diego, CA) for individual group comparisons.

## SUPPLEMENTARY MATERIALS FIGURES


