# Supplementary material for: Inhibition of the MEK/ERK pathway augments nab-paclitaxel-based chemotherapy effects in preclinical models of pancreatic cancer
Source: Oncotarget. 2017 Dec 25;9(4):5274–86. doi: 10.18632/oncotarget.23684 (PMC5797049; doi:10.18632/oncotarget.23684)
Supplement: Supplementary file 1 [file oncotarget-09-5274-s001.pdf]

Inhibition of the MEK/ERK pathway augments nab-paclitaxel-based chemotherapy effects in preclinical models of pancreatic cancer

SUPPLEMENTARY MATERIALS

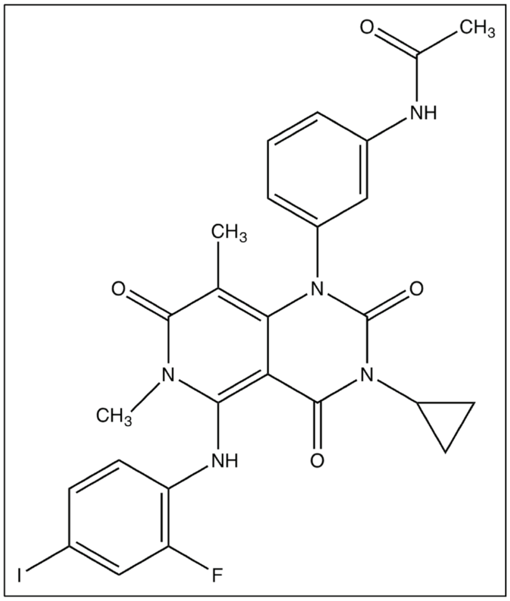

Trametinib

Supplementary Figure 1: Chemical structure of trametinib. Molecular weight: 615.39.

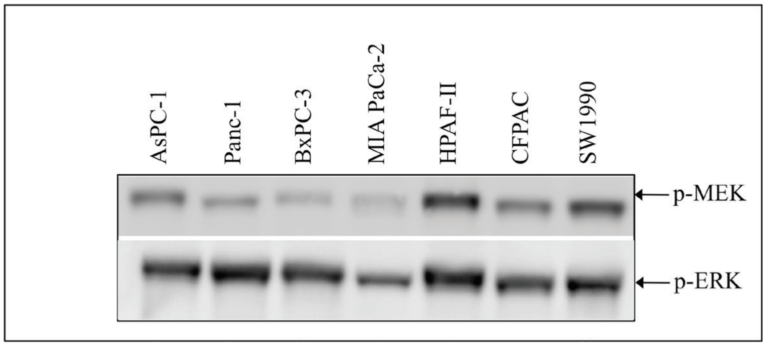

Supplementary Figure 2: Expression of phospho-MEK and phospho-ERK in PDAC cell lines. Whole cell lysates were prepared from PDAC cells, and equal amounts of total protein in precleared lysates were analyzed by immunoblotting.
